# Cutaneous Manifestations of Inborn Errors of Immunity: Clinical Clues to Immune Disorders

**DOI:** 10.3390/medicina62030581

**Published:** 2026-03-19

**Authors:** Katarzyna Napiorkowska-Baran, Maciej Pastuszczak, Maria Płocka-Karpińska, Marta Tykwińska, Paweł Treichel, Gary Andrew Margossian, Carla Liana Margossian, Agnieszka Rogalska, Rafał Czajkowski

**Affiliations:** 1 Department of Allergology, Clinical Immunology and Internal Diseases, Collegium Medicum in Bydgoszcz, Nicolaus Copernicus University, 85-168 Torun, Poland; maciej.pastuszczak@sum.edu.pl (M.P.); marta.tykwinska@cm.umk.pl (M.T.); 2 Department of Dermatology, Medical University of Silesia, 40-027 Katowice, Poland; 3 Academia Copernicana Interdisciplinary Doctoral School, Nicolaus Copernicus University, 87-100 Torun, Poland; maria.plocka@wp.pl; 4 Doctoral School of Medical and Health Sciences, Collegium Medicum in Bydgoszcz, Nicolaus Copernicus University, 85-067 Torun, Poland; treichel.pawel@gmail.com; 5 School of Medicine Faculty, New York Medical College, Valhalla, NY 10595, USA; garymargossian@gmail.com; 6 Jacobs School of Medicine and Biomedical Sciences, University at Buffalo, Buffalo, NY 14203, USA; carlamargossian@gmail.com; 7 Jan Biziel University Hospital No. 2, 85-168 Bydgoszcz, Poland; dyr.naczelny@biziel.pl; 8 Department of Dermatology and Venereology, Faculty of Medicine, Ludwik Rydygier Collegium Medicum in Bydgoszcz, Nicolaus Copernicus University, 85-094 Torun, Poland; r.czajkowski@cm.umk.pl

**Keywords:** inborn errors of immunity, primary immunodeficiencies, cutaneous manifestations, dermatologic phenotypes, recurrent skin infections, autoimmune skin diseases, atopic dermatitis, granulomatous dermatoses, neoplastic skin lesions, immunodermatology

## Abstract

*Background/Objectives*: Cutaneous manifestations of inborn errors of immunity (IEI) are among the most common and often early signs of these disorders, estimated to affect about 40% of patients with IEI, and in some cases, they provide the first diagnostic clue. Skin findings in IEI are heterogeneous and include recurrent skin infections, severe atopic dermatitis, autoimmune manifestations, as well as atypical granulomatous dermatoses, neoplastic lesions, pigmentation disorders, and changes involving hair and nails. Early recognition of these manifestations and linking them to the appropriate immunologic defect is crucial for establishing the diagnosis and initiating targeted therapy. *Methods*: This paper reviews the dermatologic phenotypes associated with IEI, with particular emphasis on a tabular classification of skin lesions corresponding to specific immunologic defects. Relevant literature was analyzed to summarize characteristic cutaneous presentations and current diagnostic approaches, highlighting the importance of interdisciplinary evaluation. *Results*: Cutaneous findings in IEI encompass a wide spectrum of infectious, inflammatory, autoimmune, and neoplastic manifestations. Systematic classification of these lesions facilitates earlier recognition of underlying immune defects and supports differential diagnosis. Dermatologic signs frequently precede systemic manifestations, making them valuable early clinical indicators of IEI. *Conclusions*: Recognition of dermatologic manifestations is critical for early diagnosis of IEI. Interdisciplinary collaboration between dermatologists, immunologists, and other specialists improves diagnostic accuracy and patient management. Current therapeutic strategies range from symptomatic treatment to targeted therapies, and personalized approaches improve prognosis and quality of life in patients with IEI.

## 1. Introduction

As the largest organ of the human body, the skin provides protection against external factors. Careful observation of the skin can offer early insight into the presence of inborn errors of immunity (IEI), formerly known as primary immunodeficiencies (PID). IEI comprises a large group of genetically determined diseases characterized by defects in the immune system. They manifest as increased susceptibility to infections, a tendency toward autoimmune diseases and malignancies, and chronic inflammation. In patients with IEI, cutaneous lesions may guide the diagnostic workup and thereby accelerate disease recognition [1,2].

Several recent reviews have summarized the spectrum and diagnostic relevance of cutaneous manifestations in IEI, including comprehensive overviews of dermatologic findings across IEI. This work builds on and extends these prior reviews by integrating recent epidemiologic evidence on the frequency and early presentation of skin changes in IEI populations, discussing specific cutaneous features as clinical indicators of underlying immune defects, providing focused analyses of atopic dermatitis within IEI contexts, and offering practical guidance for the recognition and management of dermatologic features in these syndromes [1,3,4,5].

## 2. Methodology

Structured searches of PubMed, Scopus and Web of Science databases (2000–2025) were performed. References were managed using Zotero version 7.0 (Corporation for Digital Scholarship, Vienna, VA, USA). Inclusion criteria comprised studies describing dermatologic manifestations in inborn errors of immunity with confirmed clinical or genetic diagnosis, whereas exclusion criteria included lack of dermatologic data or unclear immunologic diagnosis. A narrative synthesis approach was applied due to heterogeneity of available evidence.

This work is based on a comprehensive review of the current literature and available epidemiologic data on cutaneous manifestations accompanying immune defects. The collected information indicates that a substantial proportion of patients with IEI (40.3%) present with skin lesions, and in some of them, these lesions are even the first clinical manifestation of the disease [1]. Accurate identification of cutaneous manifestations and their classification into appropriate categories of immune defects is important because certain medications can obscure the clinical picture or even induce secondary immunodeficiency, thereby worsening the course of the underlying disease. The following sections discuss the immunologic mechanisms leading to skin lesions in IEI and present the main types of dermatologic manifestations in these syndromes. Diagnostic and therapeutic proposals are also outlined, highlighting the importance of early recognition of the cutaneous phenotype for improving patient outcomes.

The manuscript represents a comprehensive narrative review and Table 1 provides representative but clinically oriented groupings of dermatologic manifestations rather than an exhaustive catalogue.

## 3. Immunopathogenesis of Cutaneous Lesions in IEI

Immunopathogenetic mechanisms play an important role in shaping skin lesions in IEI. Understanding the interactions between immune system function, genetic defects, and the role of the skin and its structures in the immunopathogenesis of IEI helps explain the development of dermatologic symptoms, their diagnosis, and treatment.

### Mechanisms of Innate and Adaptive Immunity

The cutaneous immune response influences the pattern of dermatologic lesions in patients with IEI. Both innate and adaptive immunity determine the severity and recurrence of lesions, and the type of cellular or molecular defect translates into a specific dermatologic phenotype. For example, disruption of innate immune signaling pathways (MyD88/IRAK-4) results in recurrent and often severe bacterial infections, particularly with encapsulated organisms such as *Streptococcus pneumoniae*. In some children, the initial presentation may include eczematous or inflammatory skin lesions that resemble atopic dermatitis. Because these cutaneous manifestations can precede severe infections or occur independently of them, they may be misinterpreted as spontaneous atopic dermatoses. This misinterpretation can delay recognition of the underlying immunodeficiency, emphasizing the need for careful evaluation of early-onset or atypical dermatologic findings in the context of recurrent infections [6,7,8].

The integrity of the skin barrier, immune mechanisms, and immune surveillance in the skin depend largely on the presence of Langerhans cells (LC) and dendritic cells (DC). Their dysfunction (e.g., loss of immune tolerance or impaired migration) promotes chronic dermatoses, such as eczema and lichenification [9]. As reported by Pituch-Noworolska, Langerhans cells initiate the immune response by presenting antigens to T lymphocytes and play an important role in maintaining tolerance via regulatory T cells. Maintaining local immune tolerance by LC is crucial for skin homeostasis—loss of this tolerance may lead to uncontrolled inflammation, development of skin infections, and disruption of the microbiological balance on the skin surface [10]. Both infections and dysbiosis of the skin microbiota drive a vicious cycle of chronic inflammation. Different DC subpopulations (e.g., cDC1, cDC2) also have important functions; for example, cDC2 can migrate to lymph nodes, and their impairment can directly lead to chronic dermatoses [11].

Mutations in genes associated with IEI, such as DOCK8, WAS, NEMO, STAT3, and ADA2, lead to diverse defects of innate and adaptive immunity, resulting in different cutaneous phenotypes. For instance, the absence of pattern recognition receptors (PRR) for viral and fungal pathogens increases the risk of infections with these pathogens, while deficiencies of B and T lymphocytes favor chronic cutaneous inflammation [12]. As Cagdas et al. report, dermatologic phenotypes are, in most cases, characteristic of specific mutations, allowing many IEIs to be diagnosed based on the skin picture [13]. Thus, in patients with extensive, treatment-resistant eczema, mutations in WAS or STAT3 may be suspected [8]. Genotype–phenotype analysis facilitates diagnostics: based on the type of dermatologic lesions, clinicians can decide on targeted immunologic and genetic testing, shortening the path to diagnosis and preventing complications.

The skin microbiome and cytokine profile also influence the dermatologic phenotype in IEI. In patients with STAT3 mutations, which cause deficiency of IL-17 and IL-22 and Th17 lymphocytes, chronic fungal infections of the skin are observed [14]. It has also been found that the skin microbiome of patients with IEI is less diverse, and dominant chronic bacterial superinfections further fuel inflammation and worsen microbiota status. A characteristic combination of a specific molecular defect with a dysbiotic microbiome may generate an individual “immunologic–microbiologic signature” in a given patient, leading to severe cutaneous manifestations of IEI [15].

**Figure 1:**
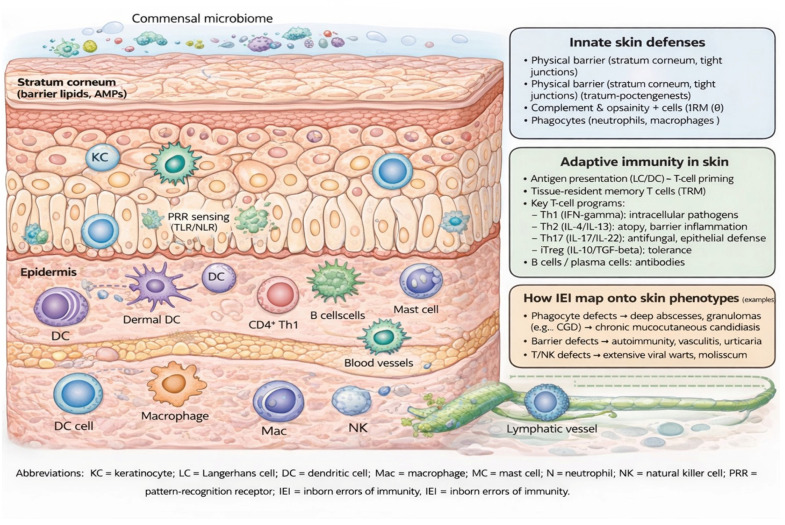
Skin Immunology—Key Components and Immune Pathways.

Knowledge of the above mechanisms of skin lesion development enables identification of patients with atypical, chronic, or recurrent dermatoses (e.g., difficult-to-eradicate fungal infections) as candidates for evaluation for IEI [16,17]. Early recognition allows prompt initiation of genetic diagnostics and appropriate causal therapy, which may prevent further cutaneous and systemic complications. The key components and immunological pathways of the skin are shown in Figure 1.

## 4. Cutaneous Manifestations in IEI

Available epidemiologic data suggest that infectious skin manifestations are the most frequent dermatologic findings in inborn errors of immunity, followed by eczema-like dermatoses, whereas autoimmune and autoinflammatory lesions are less common but often provide key diagnostic clues. The exact prevalence varies across IEI subtypes and cohorts [18].

Table 1 presents representative examples of dermatologic manifestations observed in inborn errors of immunity. The listed IEIs were selected based on the strength of documented association, their illustrative value for specific dermatologic patterns, their clinical relevance for diagnostic decision-making, and their frequency in clinical practice.

### 4.1. Infectious and Atopic Lesions

Abscesses and recurrent bacterial superinfections represent the most common infectious cutaneous manifestations observed in inborn errors of immunity (IEI), whereas chronic ulcers and perianal fistulas more often reflect underlying immune dysregulation and impaired tissue repair rather than primary atopic or allergic disease. These manifestations are particularly frequent in disorders with phagocytic defects such as chronic granulomatous disease (CGD) and in cellular immunodeficiency syndromes including hyper-IgE syndromes (HIES) and combined immunodeficiencies [19].

Chronic non-healing ulcers and perianal fistulizing disease have also been described in IEI associated with dysregulated neutrophilic inflammation and cutaneous immune dysfunction, and should prompt expanded immunologic evaluation when occurring with other systemic features of immune dysregulation [19,20,21,22].

In CGD, up to 75% of patients present with infectious skin lesions, most commonly caused by *Staphylococcus aureus* and *Corynebacterium* spp. [23]. Nearly all patients with HIES experience skin abscesses [24]. In young children with IEI, skin infections may initially be the only manifestation of immune dysfunction, which can lead to delayed or incorrect diagnosis. This underscores the need for early interdisciplinary management (dermatologist, immunologist, pediatrician) to establish the correct diagnosis.

In addition to bacterial infections, patients with defects of the Th17 pathway and impaired phagocyte function often develop recurrent fungal and viral infections of the skin and mucous membranes. Clinically, these can resemble chronic inflammatory dermatoses. If a patient with recurrent skin infections develops successive superinfections and shows no improvement despite standard dermatologic treatment, immunologic diagnostics should be expanded to evaluate for IEI. Chronic skin ulcers and perianal fistulas in children that fail to resolve with conventional therapy and coexist with frequent infections are additional red flags for IEI. Chronic and recurrent skin infections have been shown to significantly burden patients, affecting the severity of IEI and reducing quality of life [25].

Eczema and atopic dermatitis (AD) are common dermatologic manifestations in IEI, particularly in Wiskott–Aldrich syndrome and hyper-IgE syndromes [5]. Severe, recurrent, treatment-resistant eczema often leads to secondary skin superinfections. Therefore, in a young child who develops extensive eczematous lesions early in life, immunologic evaluation for an inborn error of immunity should be considered only in the presence of red flags suggestive of IEI, such as recurrent or severe infections, poor response to standard topical and systemic therapy, failure to thrive, chronic mucocutaneous candidiasis, cytopenias (e.g., thrombocytopenia), or a positive family history of primary immunodeficiency. Atopic dermatitis in the course of IEI often presents with pronounced, treatment-resistant lichenification that does not respond to standard topical glucocorticoid therapy, predisposing to further infections. In patients with such features, especially when disease control cannot be achieved despite treatment, evaluation for IEI should be considered [26]. Proper interpretation of severe, refractory eczema as a possible early manifestation of a primary immunodeficiency helps avoid unnecessary therapeutic interventions and shortens the time from symptom onset to diagnosis.

Chronic and recurrent fungal infections of the skin and mucous membranes—especially those caused by *Candida* spp. and *Aspergillus* spp.—are frequently observed in patients with IEI. This applies particularly to patients with impaired Th17 responses, for example in STAT3 mutations limiting IL-17 and IL-22 production, as well as in leukocyte adhesion deficiencies or other mutations (e.g., MUC5B gene deletion) [27]. These infections do not respond to standard treatment, tend to recur, and may occur in atypical locations—among children they should immediately raise suspicion of IEI [22]. The basis of chronic cutaneous mycoses in these patients is mainly impaired cellular immunity, particularly the lack of Th17 responses caused by defects in the IL-17/IL-22 pathway (e.g., STAT3 mutations) [27].

### 4.2. Autoimmune Disorders

Autoimmunity plays an important role in the clinical picture of IEI, especially in patients with defects of immune response regulation. The frequency of autoimmune and inflammatory manifestations in IEI reaches ~10%, and in some subgroups (children with regulatory defects, phagocytic disorders, or combined defects) exceeds 50% [28]. The presence of autoimmune manifestations is therefore an important diagnostic clue when differentiating IEI and requires appropriate monitoring for complications.

In young patients, chronic skin lesions persisting for more than six weeks—such as non-healing ulcers, recurrent rashes, blisters, or pigmentary disturbances—should prompt an expanded diagnostic workup, particularly immunologic, when these features occur in the context of immune dysregulation or other systemic manifestations [29]. Such pigmentary changes may arise from autoimmune processes or from syndromes associated with immune dysregulation, including conditions predisposing to hemophagocytic lymphohistiocytosis (HLH), pyogenic infections, or pigmentary mosaicism. Early recognition of these cutaneous signs facilitates timely identification of the underlying immunologic abnormalities [28,29].

Given the wide range of autoimmune symptoms that can coexist in a single patient with IEI, careful skin assessment together with evaluation of other organ systems is diagnostically important. Some cutaneous manifestations of autoimmunity—such as chronic urticaria, ulcers, or depigmentation—appear in IEI patients even before the full clinical picture of the disease develops. In children with chronic inflammatory skin lesions, other features of immune dysregulation are also common, such as food intolerances or failure to thrive [30].

In several autoinflammatory IEI, urticaria-like lesions correspond histopathologically to neutrophilic dermatoses rather than classic mast-cell–mediated urticaria, and should be distinguished from urticarial vasculitis using biopsy and complement testing. Skin biopsies in neutrophilic dermatoses usually show dense neutrophil-dominated dermal infiltrates, frequently accompanied by leukocytoclasia but lacking considerable mast-cell degranulation or dermal edema. On the other hand, mast cells, lymphocytes, and eosinophils make up the majority of the sparse perivascular infiltrates that accompany superficial cutaneous edema in classic urticaria. These distinctions are crucial in differentiating neutrophilic urticarial dermatosis from mast-cell-mediated urticaria and from urticarial vasculitis, which is distinguished histologically by complement deposition and leukocytoclastic vasculitis [1,31].

Autoimmune skin changes in IEI result mainly from defects in self-tolerance mechanisms (e.g., impaired regulatory T-cell function), whereas autoinflammatory cutaneous manifestations arise from dysregulated innate immune activation and inflammasome-driven pathways. Such manifestations have been frequently reported in association with mutations in immune-regulatory genes such as IRF4, TLR7, or UNC93B1. For example, the occurrence of cutaneous manifestations of systemic lupus erythematosus and other autoimmune diseases in children has been observed to correlate with mutations in the ERN1 gene (encoding an endoplasmic reticulum stress sensor). Loss of immune tolerance and excessive activation of pro-inflammatory cytokines lead to skin damage, resulting in ulcers, blisters, and other chronic lesions [32]. The mechanisms of cutaneous manifestations in IEI are complex and include genetically determined defects of immune tolerance leading to autoimmunity, as well as primary dysregulation of innate immune pathways driving autoinflammatory skin disease. Therefore, integration of dermatologic and immunologic expertise is crucial when assessing these patients [33].

The spectrum of autoimmune cutaneous manifestations in IEI is very broad. Chronic inflammatory skin lesions (persistent rashes, blistering lesions, non-healing ulcers) may reflect either autoimmune processes (adaptive immune dysregulation) or autoinflammatory mechanisms (innate immune hyperactivation), which represent distinct pathogenetic entities [34]. Studies have shown that skin symptoms often precede other IEI manifestations—for example, in the observation by Costa-Carvalho et al., in ~32% of patients skin lesions appeared before an IEI diagnosis was established [35]. To confirm the autoimmune nature of skin symptoms, biopsy of the lesion with immunohistochemical assessment is recommended. This helps distinguish dermatoses resulting from autoimmune disease from lesions caused by chronic infection or drug effects [36]. Accurate determination of whether cutaneous lesions are autoimmune or autoinflammatory in origin is crucial for selecting appropriate targeted therapy and for influencing the patient’s prognosis.

Any chronic dermatosis of unclear etiology, such as pemphigus, scleroderma, or lupus, in children should prompt clinicians to consider an immunologic background, including an inborn immune defect. In most of these cases, further diagnostics are required (skin biopsy with histopathologic and immunofluorescence examination), which can reveal characteristic inflammatory infiltrates (e.g., immunoglobulin deposits, granulomas) [37]. After confirming the autoimmune character of the cutaneous lesions, immunologic and genetic evaluation should be expanded to identify the specific immune disorder. Such a comprehensive approach yields more effective causal treatment. Because clinical pictures of primary and secondary autoimmune dermatoses can overlap and available diagnostic methods have limitations, autoimmune skin manifestations in IEI may be overlooked. Education of medical staff (dermatologists, immunologists, pediatricians) on the recognition of dermatologic manifestations of immunodeficiency is therefore necessary [38].

In summary, autoimmune skin lesions are typical of many immunologic disorders in patients with IEI. They encompass a very wide spectrum of symptoms—from urticaria and rashes, through blistering lesions and ulcers, to pigmentary changes or scleroderma-like features—and always require comprehensive diagnostics to establish the correct diagnosis.

### 4.3. Neoplastic and Premalignant Skin Lesions

Patients with IEI are at increased risk not only of infections but also of developing malignancies, including skin cancers. This is contributed to by chronic antigenic stimulation (e.g., by oncogenic viruses) and inadequate immune surveillance of neoplastic transformation [39]. The most common neoplastic dermatologic manifestations in IEI include skin carcinomas (especially squamous cell carcinoma and basal cell carcinoma) and cutaneous lymphomas [40,41]. For example, in WHIM syndrome (CXCR4 mutation), chronic cutaneous HPV infection leads to warts and may increase the risk of cutaneous squamous cell carcinoma and cervical dysplasia [42,43]. In DOCK8 deficiency, associated with severe immunodeficiency, frequent occurrence of cutaneous T-cell lymphomas and aggressive squamous cell carcinomas has been described, representing one of the main causes of death in these patients [44,45]. Dyskeratosis congenita—a rare telomere disorder—manifests with leukoplakia of mucous membranes and skin, which is a premalignant condition and may precede the development of squamous cell carcinoma [46]. Cartilage-hair hypoplasia (McKusick disease) in turn increases the risk of basal cell carcinoma [47].

It should be emphasized that chronic viral skin infections in IEI patients (e.g., caused by oncogenic HPV types or HHV8) may lead to malignancy. An example is Kaposi sarcoma (associated with HHV8) described in patients with severe T-cell deficiencies [48]. Chronic inflammation and granulomas may also predispose to carcinogenesis. Therefore, any chronic, atypical skin lesions in IEI patients—especially verrucous, nodular, or non-healing lesions—require dermoscopic and histopathologic evaluation for malignant transformation. Early detection and treatment of skin cancers in IEI patients improves prognosis. Table 1 summarizes the most important neoplastic and premalignant skin lesions described in IEI together with the corresponding syndromes (e.g., squamous cell carcinoma—WHIM syndrome, EVER1/EVER2 syndrome; cutaneous lymphomas—TACI deficiency; leukoplakia—dyskeratosis congenita, etc.) [46,47].

### 4.4. Granulomatous Dermatoses and Skin Nodules

Granulomatous lesions are characteristic manifestations of some IEIs, particularly those associated with chronic macrophage activation and granuloma formation. Granulomatous infiltrates in the skin can mimic sarcoidosis or tuberculosis. However, in IEI patients they often occur in the absence of an identifiable infectious agent (so-called nonspecific granulomas). For example, in chronic granulomatous disease (CGD) disseminated granulomas of the skin and internal organs have been described, caused by dysfunctional phagocytes unable to eliminate microorganisms-this leads to organization of chronic inflammation in the form of a granuloma 3/15/2026 10:45:00 AM. Granulomatous skin lesions are also found in some patients with common variable immunodeficiency (CVID). They may resemble sarcoidosis and often coexist with autoimmune cytopenias. In ataxia-telangiectasia (ATM gene defect) and some primary neutrophil disorders, skin granulomas have also been described, sometimes with features of a pyogenic granuloma.

Nodular dermatoses also include various inflammatory infiltrates and nodules that do not have classic granuloma histology but clinically present as subcutaneous nodules. They may occur, for example, in autoinflammatory syndromes associated with inflammasome mutations. An example is Blau syndrome (NOD2 mutation), in which papular-nodular sarcoid-like rashes are characteristic [49]. Another example is the familial cold autoinflammatory syndrome PLAID (PLCG2 mutation), in which cold exposure triggers generalized urticaria and a papular rash that can leave infiltrated lesions. In immunodeficiencies with an autoinflammatory component (e.g., APLAID, PLCG2 mutation), recurrent inflammatory nodules and erythematous patches are observed [50]. Subtle subcutaneous nodules (panniculitis) may also appear in immunodeficiencies associated with inflammation of adipose tissue. For instance, such lesions have been described in OTULIN deficiency (ORAS/otulipenia syndrome) [51].

Granulomatous lesions and skin nodules in a patient with IEI always require differentiation from infections (tuberculosis, atypical mycobacteria, deep mycoses) and sarcoidosis-spectrum diseases. Detection of granulomas on histopathologic examination of the skin in an immunocompromised patient should prompt a search for immune defects (e.g., CGD, syndromes with STAT1 GOF mutations, NOD2 mutations, etc.). Table 1 provides examples of IEIs presenting with granulomatous lesions (e.g., Job syndrome/AD-HIES, cartilage-hair hypoplasia) and related nodular dermatoses [50,52].

### 4.5. Neutrophilic and Pustular Dermatoses

Some inborn errors of immunity present with cutaneous manifestations in the form of neutrophilic dermatoses. This term encompasses lesions dominated by neutrophilic infiltrates, often appearing as erythematous patches, nodules, or pustules. Classic neutrophilic dermatoses include Sweet syndrome (acute febrile neutrophilic dermatosis) and pyoderma gangrenosum. In IEI patients, lesions analogous to Sweet syndrome or pyoderma gangrenosum have been described. For example, in Majeed syndrome (LPIN2 mutation)—an autoinflammatory disease with recurrent osteomyelitis—Sweet syndrome occurs, manifesting with fever and painful erythematous plaques on the skin [53,54]. In MHC class I deficiency (mutations in TAP1, TAP2, TAPBP), chronic skin ulcers resembling pyoderma gangrenosum have been observed [55].

Pustular skin lesions-sterile pustules and generalized pustular eruptions-may occur in IEIs associated with dysregulation of IL-1 and IL-36 cytokine pathways. An example is IL36RN deficiency (DITRA), which leads to generalized pustular psoriasis [56]. Similarly, AP1S3 mutations (AP1S3-PIA syndrome) result in difficult-to-treat palmoplantar pustular psoriasis [57]. PAPA syndrome (PSTPIP1 mutation) is characterized by recurrent sterile abscesses and lesions resembling pyoderma gangrenosum [58,59]. Importantly, chronic sterile skin abscesses are also a characteristic feature of chronic granulomatous disease—some CGD patients develop recurrent pustules and abscesses despite the absence of culturable pathogens. These abscesses are considered sterile based on repeated negative bacterial, fungal, and mycobacterial cultures, as well as molecular testing in some studies. However, we cannot completely exclude the possibility that some abscesses may be caused by obscure or difficult-to-culture organisms [23,59,60]. Immunodeficiencies with impaired neutrophil recruitment, such as hyper-IgE syndrome, can also manifest with chronic pustular lesions due to dysregulation of the cutaneous inflammatory response.

Diagnosis of a neutrophilic dermatosis in an IEI patient requires exclusion of infection (as these lesions can mimic purulent bacterial infections) and collaboration with a rheumatologist, because similar symptoms occur in autoinflammatory diseases. The tabular summary (Table 1) includes selected IEIs from this spectrum—such as PAPA syndrome, IL-1 deficiencies (DIRA) IL-36 deficiencies (DITRA), and CGD together with their skin manifestations in the form of pustules and ulcers [59,61].

### 4.6. Disorders of Skin Pigmentation

Abnormalities of skin and hair pigmentation constitute another group of manifestations accompanying some immunologic defects. They can include generalized hypopigmentation (partial albinism) as well as focal hypo- or hyperpigmentation. A classic example is Chediak–Higashi syndrome, characterized by partial albinism (light skin, silvery hair) resulting from mutations in the LYST gene, leading to defective melanosome granule formation [62,63]. Similarly, Griscelli syndrome (RAB27A mutation) features hypopigmented hair (silvery, metallic sheen) and recurrent infections. In some patients, the unusual hair color is the first sign [64,65].

Another example of a pigmentary disorder is X-linked reticulate pigmentary disorder (XRPD, POLA1 mutation), in which boys present with characteristic reticulate hyper- and hypopigmentation of the skin, often accompanied by recurrent infections [58]. Dyskeratosis congenita, in addition to leukoplakia, also causes a triad of cutaneous findings: reticulated skin pigmentation, nail dystrophy, and leukoplakia. These pigmentary changes often appear already in childhood [66]. Café-au-lait macules (light-brown spots) are described, among others, in Nijmegen breakage syndrome (NBN mutation) and ICF syndrome (DNMT3B mutation) and may draw the clinician’s attention to genomic instability or methylation defects in these diseases [67,68].

Pigmentary disorders also include mosaic depigmentation in some X-linked diseases, such as incontinentia pigmenti (IKBKG/NEMO gene, classified among IEIs), which progresses through stages from vesicles to verrucous lesions to hyperpigmentation along the lines of Blaschko. In Wiskott–Aldrich syndrome (WAS), acquired depigmentation resembling vitiligo has also been reported, most likely resulting from autoimmunity against melanocytes.

Pigmentary manifestations of IEI are not only a cosmetic issue—they often coexist with severe immune disorders (for example, albinism in Chediak–Higashi syndrome is associated with severe infections and hemophagocytosis). Any unexplained pigmentary abnormality in a child—especially if accompanied by infections, dysmorphic features, or hematologic symptoms—should prompt evaluation for an inborn immune defect. Table 1 presents selected syndromes with pigmentation abnormalities in the course of IEI, including silver hair syndromes (Chediak–Higashi, Griscelli) and other immunologic genodermatoses (dyskeratosis congenita, XRPD) together with the relevant genes [62,69].

### 4.7. Changes in Skin Appendages: Hair and Nails

Skin appendages, such as hair and nails, can also reveal signs of inborn immunodeficiencies. In some patients, abnormalities of hair (e.g., structure or color) and nails provide an important diagnostic clue. For example, in Netherton syndrome (SPINK5 mutation) hair shows the characteristic “bamboo hair” structure (trichorrhexis invaginata) and breaks easily [70]. This is accompanied by erythroderma and atopic dermatitis from infancy. Another example is the winged-helix nude syndrome (FOXN1 mutation), which manifests as congenital lack of hair (atrichia) and thymic hypoplasia. In such newborns, generalized alopecia may be the first clue to severe immunodeficiency [71,72]. In Omenn syndrome (mutations in RAG1/2, IL7R, and others), generalized exfoliative erythroderma and hair loss are observed as early manifestations of the underlying SCID phenotype. In DOCK8 deficiency (AR-HIES), patients’ hair may be brittle and sparse, and some patients develop premature graying for unclear reasons [73,74]. Interestingly, IRF4 mutations (immunodeficiency with atopic features) may manifest in childhood precisely as premature graying of hair accompanied by atopy and susceptibility to infections [73].

Nail changes can also suggest IEI. Nail dystrophy, typically presenting as thinning and fragility of the nails, can be a leading sign of dyskeratosis congenita [75,76]. In these patients, in addition to nail changes there are pigmentary abnormalities and a predisposition to malignancies. Nail fragility and deformities have also been described in ectodermal dysplasia with immunodeficiency. For example, in NEMO syndrome (IKBKG mutations) and NF-kB1A deficiency where nail abnormalities coexist with recurrent skin infections and atopic eczema [77]. Another example is Clericuzio syndrome (poikiloderma with neutropenia, USB1 mutation), in which poikiloderma, neutropenia, and nail dystrophy are present [78,79]. In chronic mucocutaneous candidiasis (e.g., APECED due to AIRE mutation, or IL-17 pathway deficiencies), the nail plates may also be destroyed secondarily due to onychomycosis—recurrent onychomycosis in a child should raise immunologic suspicion.

Hair and nail abnormalities rarely occur in isolation—most often they coexist with other skin findings (e.g., infections, ichthyosis, erythroderma). Nevertheless, their presence should prompt a search for links with IEI. Table 1 provides examples of hair and nail changes in IEI, including Netherton syndrome (bamboo hair), ectodermal dysplasias with immunodeficiency (brittle nails), and woolly hair syndromes (curly, woolly hair in tricho-hepato-enteric syndrome) [3,70].

### 4.8. Vascular Lesions and Cutaneous Vasculopathies

Vascular diseases of the skin, including vasculitis, can also be manifestations of inborn immune defects. In IEI patients, both leukocytoclastic cutaneous vasculitis and more complex vasculopathies leading to ischemic ulcers are observed. For example, ADA2 deficiency (also known as PACNS or mPAN) causes systemic inflammation of small and medium vessels, and patients often have livedo racemosa and skin ulcers resulting from vasculitis [80]. In children with ADA2 deficiency, recurrent macular rashes, necrotic crusts on the skin, and lesions resembling panniculitis have been described. Another example is DNASE1L3 deficiency, in which necrotizing cutaneous vasculitis and ulcers occur already at a young age.

Chronic skin ulcers may also indicate inborn errors of metabolism with immunologic consequences. For example, prolidase deficiency (PEPD mutation) results in severe, difficult-to-heal ulcers on the feet and lower legs [81]. In such patients, ulcers are the result of both vascular factors and infections. Recognition of the molecular cause (enzyme deficiency) makes it possible to introduce glycine and proline supplementation, which can alleviate lesions. In turn, constitutive activation of HCK kinase (HCK mutation, described by Kanderová et al.) leads to severe, early leukocytoclastic cutaneous vasculopathy and lung damage [82,83]. In the clinical picture of these patients, petechiae, ulcers, and systemic signs of vasculitis predominate.

Cutaneous vascular lesions also include telangiectasias (for example, ataxia-telangiectasia is characterized by dilation of small vessels on the conjunctiva and facial skin) as well as Raynaud phenomenon and livedo reticularis, seen in some autoinflammatory syndromes. Although telangiectasias themselves are benign, in the context of ataxia-telangiectasia they indicate a serious immunologic and neurologic defect (ATM mutation).

Recognition of vasculitis or chronic cutaneous vasculopathy in a young patient should raise suspicion of IEI, especially if other immunologic abnormalities coexist (e.g., humoral immune deficiency in ADA2). The diagnostic workup should include skin biopsy with histopathologic evaluation of vessels (searching for fibrinoid necrosis and immune-complex deposits) and genetic testing for known monogenic inflammatory syndromes. Table 1 summarizes examples of vascular lesions in IEI, including ulcers and vasculitis in ADA2 deficiency and prolidase deficiency syndrome [80,84].

## 5. Diagnostics and Clinical Management

### 5.1. Importance of the Cutaneous Phenotype

Accurate assessment of the cutaneous phenotype is important for the early recognition of characteristic lesions, which can prompt timely referral for appropriate diagnostic evaluation and management in IEI. A holistic approach is required, combining clinical skin assessment with laboratory and genetic tests and establishing individualized management algorithms. A detailed analysis of the dermatologic phenotype provides important support for the diagnostic process and should take into account all categories of lesions described above (infectious, allergic, autoimmune, neoplastic, structural, and others). Importantly, in about half of patients, skin lesions are among the first signs of IEI; therefore, the skin can be treated as a “window” to diagnosing these diseases. Taking a thorough dermatologic history and documenting lesions (e.g., photographs) help capture the dynamics of the disease process, for example, progression from atopic dermatosis to recurrent deep infections or the appearance of new lesions (blisters, tumors) during the course of the disease. Such a change in the clinical picture may suggest progression of the immune defect or the emergence of complications (e.g., malignancy) [30].

Skin biopsy can also provide useful diagnostic information in some circumstances. Histopathologic examination might help differentiate infectious lesions from inflammatory or autoimmune dermatoses, revealing features such as granulomatous inflammation, vasculitis, neutrophilic infiltrates, or malignant transformation. Therefore, biopsy is a valuable supplementary diagnostic tool for patients with uncharacteristic, persistent, or treatment-resistant cutaneous lesions, especially when read in conjunction with immunologic and genetic data [2].

In differential diagnosis, team collaboration is extremely important. A dermatologist, clinical immunologist, allergist, and often also a rheumatologist and hematologist should jointly evaluate a patient suspected of IEI. The skin can provide visible clues to underlying immune dysfunction, highlighting the importance of vigilance and comprehensive diagnostic evaluation by clinicians [85,86]. For example, chronic candidiasis of the skin and nails in a young person should prompt evaluation for defects in the IL-17 pathway (STAT1, IL17F, IL17RA mutations), whereas extensive oral leukoplakia should prompt evaluation for dyskeratosis congenita or APECED. It is crucial to connect even seemingly distant symptoms (e.g., recurrent lung infections and skin lesions such as urticaria or vitiligo) and consider a shared immunologic background. Literature data indicate that patients with cutaneous manifestations experience longer diagnostic delays—probably because primary care physicians do not sufficiently associate these symptoms with IEI [87,88]. Improving awareness and knowledge of dermatologic phenotypes of IEI can significantly shorten the time to diagnosis.

### 5.2. Therapeutic Algorithms

Management of skin lesions in IEI requires individualization based on careful analysis of the dermatologic phenotype and precise identification of the type of immunodeficiency. In order to diagnose potential inborn defects of immunity, genetic testing is essential. When clinical signs and preliminary laboratory tests indicate an underlying immunological deficiency, targeted gene panels or whole-exome sequencing can confirm the molecular diagnosis and guide treatment decisions [3]. The cornerstone is causal treatment of IEI, which often leads to improvement of cutaneous symptoms as well.

The choice of causal therapy (e.g., immunoglobulin replacement, antibiotic prophylaxis, or hematopoietic stem cell transplantation—HSCT) depends on the molecular basis of the disease and the patient’s overall condition. For example, in patients with severe recurrent skin infections or refractory atopic dermatitis in the setting of SCID, Wiskott–Aldrich syndrome, DOCK8 deficiency, or CGD, early qualification for allogeneic HSCT often leads to resolution of skin lesions and improvement of immune function [89]. However, potential transplant complications must be remembered (including graft-versus-host disease, GVHD), which may also manifest in the skin and require intensive immunosuppressive treatment.

Symptomatic treatment targeted to specific lesions is also crucial. Interventions include, among others: topical and systemic antibiotics (for bacterial infections), antifungal agents (chronic cutaneous candidiasis), retinoids and keratolytics (extensive HPV warts), topical calcineurin inhibitors and immunomodulators (chronic eczema, lichenification), phototherapy (diffuse eczematization, pruritus), and specialist dressings and skin-protection measures (chronic ulcers). In patients with autoimmune disorders, systemic immunosuppression (e.g., systemic corticosteroids, mycophenolate, cyclosporine) may be necessary to control severe skin lesions. In many IEI patients with concomitant atopic dermatitis, biologics such as anti-IgE therapy (omalizumab) or dupilumab (IL-4Rα antibody) can serve as bridge therapy before immune reconstitution. Dupilumab has been shown to be safe and highly effective in patients with DOCK8, STAT3, or CARD11 mutations, achieving marked reduction of lesion severity and improved quality of life without serious infections [90,91,92]. Other pathway-targeted therapies are also increasingly relevant, including JAK inhibitors for interferonopathies or STAT3-related atopic disease, and IL-1 antagonists in autoinflammatory syndromes, offering symptom control when conventional therapies fail [93,94,95].

HPV-associated warts in IEI often present atypically: they can be multiple, widespread, recalcitrant, and with unusual morphology, which should prompt evaluation for underlying immunodeficiency. Treatment may require a combination of local therapies (cryotherapy, topical cidofovir, imiquimod), systemic antivirals in selected cases, and consideration of immune reconstitution (e.g., HSCT in severe T-cell defects) to achieve long-term control [96,97,98].

Intensive supportive treatment should be provided in parallel: avoidance of aggravating factors (contact allergens, UV exposure in patients with a predisposition to malignancy), prevention of skin infections (e.g., antiseptic baths, periodic decolonization of *Staphylococcus aureus*), and patient and caregiver education on skin care. Long-term immunoglobulin administration (IVIG/SCIG) not only reduces the frequency of systemic infections but also improves skin status, decreasing, for example, the frequency of boils and abscesses [9,99].

Regular assessment of intervention effectiveness is essential: improvement in skin status often reflects successful immunologic treatment, whereas lack of improvement or emergence of new lesions (e.g., warts, ulcers, tumors) may signal the need to modify management or detect new complications (e.g., post-transplant lymphoproliferation, secondary skin cancer). Optimal management therefore combines causal therapy with coordinated local and systemic dermatologic treatment, guided by lesion-specific characteristics and molecular diagnosis. Early recognition of atypical skin manifestations, careful sequencing of therapies (topical vs. systemic, pre- vs. post-immune reconstitution), and awareness of biologic options can improve outcomes in patients with IEI.

## 6. Conclusions

Skin lesions in IEI are common, diverse, and provide valuable clinical clues to the immunologic basis of the disease. Correct interpretation of these manifestations (infectious, atopic, autoimmune, neoplastic, and others) enables earlier recognition of IEI, which is crucial for initiating appropriate therapy before irreversible complications develop. The patient’s dermatologic phenotype can directly point to a specific immunologic defect. Thus, the skin constitutes a valuable “barometer” of the immune system’s status.

Neglecting atypical and treatment-resistant dermatoses in children and young adults may lead to significant diagnostic delays. Skin lesions should be included among the early warning signs of primary immunodeficiencies. Interdisciplinary collaboration—especially between dermatologists and immunologists—is essential for appropriate interpretation of symptoms and selection of tests. The development of modern therapies, including biologic drugs, creates new opportunities to treat burdensome cutaneous manifestations of IEI and improve patients’ comfort and quality of life.

It is important to recognize a few of the literature’s existing shortcomings. There is still considerable variation in the number of cohorts, diagnostic standards, and reporting guidelines among published data on dermatologic symptoms of inborn defects of immunity. Direct comparison of IEI subtypes may be limited by the retrospective nature of many research and their emphasis on specific traits. In order to better establish genotype–phenotype associations and maximize targeted therapy options in immunodermatology, prospective, multicenter studies combining dermatologic assessment with molecular diagnostics should be the focus of future research [18,31].

Cutaneous symptoms are an important element of the patient’s immunologic phenotype. Recognizing and understanding them in the context of IEI translates into faster diagnostics, targeted treatment, and better prognosis. Dissemination of knowledge about dermatologic manifestations of inborn immune defects should contribute to improved detection of these diseases. Further research in the dynamically developing field of immunodermatology will support the development of more effective, personalized therapeutic methods, combining advances in immunology and dermatology for the benefit of patients.

## Figures and Tables

**Table 1:** Cutaneous manifestations of inborn errors of immunity.

CLINICAL DOMAIN	DERMATOLOGIC MANIFESTATIONS	REPRESENTATIVE IEI (GENETIC GROUPS)	DIAGNOSTIC CLUES/RECOMMENDED TESTS
**INFECTIOUS MANIFESTATIONS**			
Bacterial infections	Recurrent abscesses, boils, deep tissue infections, impetigo-like lesions, ecthyma, cellulitis	Chronic granulomatous disease (CYBB, NCF1, NCF2); STAT3 deficiency (STAT3); leukocyte adhesion deficiency (ITGB2)	Bacterial culture; dihydrorhodamine oxidative burst assay; nitroblue tetrazolium test
Viral infections	Extensive viral skin infections (HPV warts, HSV-1/2 infections, VZV infections, molluscum contagiosum virus lesions)	DOCK8 deficiency (DOCK8); GATA2 deficiency (GATA2); T-cell deficiencies	Viral PCR from lesions; flow cytometry
	Wild-type rubella virus detected in granulomatous skin lesions	Ataxia-telangiectasia (ATM)	Rubella virus PCR from tissue; rubella serology
Fungal infections	Chronic mucocutaneous candidiasis; invasive fungal disease	STAT1 gain-of-function; IL-17 pathway defects (IL17RA, IL17F); CARD9 deficiency (invasive fungal infections)	Fungal culture; Candida PCR; evaluation of Th17 cell responses
Opportunistic infections	Disseminated viral or fungal infections with skin involvement	Severe combined immunodeficiency (IL2RG, RAG1, RAG2); DOCK8 deficiency	PCR; flow cytometry
	Disseminated tuberculosis infection or atypical mycobacterial infection)	IFN-γ/IL-12 pathway defects (IL12RB1, IFNGR1, IFNGR2), severe combined immunodeficiency (IL2RG, RAG1, RAG2)	Mycobacterial culture; PCR from skin lesions for Mycobacterium species; IFN-γ pathway functional testing, Ziehl–Neelsen staining
	Necrotic ulcers, nodules, or abscesses caused by opportunistic bacteria (e.g., ecthyma gangrenosum due to Pseudomonas aeruginosa, cutaneous nocardiosis)	Chronic granulomatous disease (CYBB, NCF1, NCF2); DOCK8 deficiency	Bacterial culture from lesions; Gram stain; microbiologic identification of opportunistic pathogens
**IMMUNE DYSREGULATION**			
Severe eczema with recurrent infections	Early-onset severe eczema with recurrent skin infections	DOCK8 deficiency; Wiskott–Aldrich syndrome (WAS); PGM3 deficiency	Serum IgE level; eosinophil count; complete blood count with differential
Severe gingivitis/periodontal disease	Recurrent or early-onset gingivitis, oral ulcers, periodontal tissue destruction	Congenital neutropenias (ELANE, HAX1, G6PC3); leukocyte adhesion deficiency (ITGB2)	Absolute neutrophil count; neutrophil function assays
**AUTOIMMUNE DERMATOSES**			
Psoriasis, vitiligo, alopecia areata	Psoriatic plaques; vitiligo patches; alopecia areata–like foci	Common variable immunodeficiency (TNFRSF13B); selective IgA deficiency; FOXP3 deficiency	ANA autoantibodies; serum immunoglobulin levels
Cutaneous lupus erythematosus/lupus-like lesions reported in CVID/photosensitive dermatoses	Photosensitive plaques, discoid lesions, telangiectasia	Complement deficiencies (C1Q, C2, C4); DNASE1L3 deficiency; ataxia-telangiectasia (ATM); Nijmegen breakage syndrome (NBN)	Direct immunofluorescence; complement level assessment; ANA autoantibodies; phototesting
**AUTOINFLAMMATORY/URTICARIA-LIKE DISORDERS**			
Urticaria-like eruptions	Neutrophilic dermatoses mimicking urticaria	PLCG2-associated disorders (APLAID/PLAID), **NLRP3-related cryopyrin-associated periodic syndromes (CAPS spectrum, e.g.,** FCAS, MWS—**multiple overlapping phenotypes histopathologically representing neutrophilic dermatosis)**	CRP and ESR levels; complement testing
**GRANULOMATOUS AND INFLAMMATORY LESIONS**			
Granulomatous lesions	Chronic ulcers; inflammatory nodules	Chronic granulomatous disease (CYBB); common variable immunodeficiency (TNFRSF13B); DNA repair disorders—ataxia-telangiectasia (ATM), Nijmegen breakage syndrome (NBN deficiency)	Microbiologic cultures for mycobacteria and fungi; imaging of deep tissue involvement
**MALIGNANT SKIN LESIONS**			
Squamous cell carcinoma (SCC)	Early-onset or aggressive cutaneous SCC	DOCK8 deficiency; WHIM syndrome (CXCR4)	Dermoscopy; HPV typing
Basal cell carcinoma (BCC)	Multiple or early-onset BCC	Cartilage-hair hypoplasia (RMRP)	Dermoscopy
Dermatofibrosarcoma protuberans	Slowly enlarging cutaneous tumor	Adenosine deaminase deficiency (ADA)	Dermoscopy, imaging of soft tissue mass
Epidermodysplasia verruciformis	Persistent HPV-associated skin lesions with SCC risk	EVER1/EVER2 defects (TMC6, TMC8)	Dermoscopy, HPV typing
**DEVELOPMENTAL/KERATINIZATION DISORDERS**			
Congenital ichthyosis/ichthyosiform dermatitis	Early-onset scaling, erythroderma, atopy, recurrent infections	Netherton syndrome (SPINK5); DSG1 deficiency; filaggrin variants (FLG)	Trichoscopy for hair shaft abnormalities; serum IgE level
Palmoplantar hyperkeratosis/keratinization disorders	Hyperkeratosis of palms and soles, scaling	NEMO-associated ectodermal dysplasia (IKBKG); CARD14-associated disease; Netherton syndrome (SPINK5); DSG1 deficiency; IL36RN deficiency; AP1S3 deficiency	Dermoscopy, evaluation of associated infections or inflammatory dermatoses
**DISORDERS WITH HAIR AND NAIL INVOLVEMENT**			
Hair and nail abnormalities	Alopecia, brittle hair, nail dystrophy	APECED (AIRE); STAT3 deficiency, NFKB2 deficiency, NEMO-associated ectodermal dysplasia (IKBKG)	Trichoscopy; microscopy
Sparse hair	Short, sparse hair	Cartilage-hair hypoplasia (RMRP)	Hair shaft microscopy
Congenital alopecia with immunodeficiency	Generalized alopecia	FOXN1 deficiency	Evaluation of thymic function
Syndromic hair and nail abnormalities	Hair and nail changes with congenital anomalies	CHARGE syndrome (CHD7)	Clinical syndromic assessment
**VASCULAR/PURPURA/CYTOPENIA-RELATED LESIONS**			
Petechiae and purpura	Thrombocytopenia-related lesions	Wiskott–Aldrich syndrome (WAS)	Platelet count; Mean Platelet Volume assessment
Vasculopathy	Livedo racemosa, ulcerations	ADA2 deficiency (CECR1/ADA2), STING-associated vasculopathy (TMEM173)	ADA2 enzyme activity testing, interferon signature testing
Cytopenia-associated purpura	Petechiae with immune dysregulation	LYN gain-of-function mutation	Complete blood count with differential

Bold text highlights features that are critical for differentiating between specific types of cutaneous manifestations.

## Data Availability

No new data were created or analyzed in this study. Data sharing is not applicable to this article.

## Abbreviations

The following abbreviations are used in this manuscript:

AD-HIES	Autosomal Dominant Hyper-IgE Syndrome
ADA2	Adenosine Deaminase 2
AIRE	Autoimmune Regulator
AP1S3	Adaptor Related Protein Complex 1 Sigma 3
APECED	Autoimmune Polyendocrinopathy–Candidiasis–Ectodermal Dystrophy
APLAID	Autoinflammatory PLCG2-associated Immune Dysregulation
AR-HIES	Autosomal Recessive Hyper-IgE Syndrome
ATM	Ataxia Telangiectasia Mutated
B cells	B lymphocytes
BCC	Basal cell carcinoma
CARD11	Caspase Recruitment Domain-containing protein 11
cDC1	conventional Dendritic Cell type 1
cDC2	conventional Dendritic Cell type 2
CGD	Chronic Granulomatous Disease
CVID	Common Variable Immunodeficiency
CXCR4	C-X-C Chemokine Receptor 4
DC	Dendritic cells
DIRA	Deficiency of the IL-1 Receptor Antagonist
DITRA	Deficiency of the IL-36 Receptor Antagonist
DNASE1L3	Deoxyribonuclease 1 Like 3
DNMT3B	DNA Methyltransferase 3B
DOCK8	Dedicator of Cytokinesis 8
ERN1	Endoplasmic Reticulum to Nucleus Signaling 1
EVER1/EVER2	Epidermodysplasia Verruciformis 1 and 2 (syndrome)
FOXN1	Forkhead Box N1
GOF	Gain of function
GVHD	Graft-Versus-Host Disease
HCK	Hemopoietic Cell Kinase
HHV8	Human Herpesvirus 8
HIES	Hyper-IgE Syndrome
HLH	Hemophagocytic lymphohistiocytosis
HPV	Human Papillomavirus
HSCT	Hematopoietic Stem Cell Transplantation
HSV	Herpes simplex virus
ICF	Immunodeficiency, Centromeric Instability and Facial anomalies syndrome
IEI	Inborn Errors of Immunity
IFN-γ	Interferon gamma
IgE	Immunoglobulin E
IKBKG	Inhibitor of NF-κB Kinase Gamma
IL-1	Interleukin-1
IL-12	Interleukin 12
IL-17	Interleukin-17
IL-22	Interleukin-22
IL-36	Interleukin-36
IL-4Rα	Interleukin-4 Receptor alpha
IL7R	Interleukin-7 Receptor
IRAK4	Interleukin-1 Receptor-Associated Kinase 4
IRF4	Interferon Regulatory Factor 4
IVIG	Intravenous Immunoglobulin
JAK	Janus Kinase
LC	Langerhans cells
LPIN2	Lipin 2 (Phosphatidate Phosphatase)
LYST	Lysosomal Trafficking Regulator
mPAN	Medium-size vessel Polyarteritis Nodosa (monogenic PAN)
MHC (class I)	Major Histocompatibility Complex class I
MyD88	Myeloid Differentiation Primary Response 88
NBN	Nibrin
NEMO	NF-κB Essential Modulator
NF-κB1A	NF-κB Inhibitor Alpha (IκBα, gen NFKBIA)
NOD2	Nucleotide-binding Oligomerization Domain-containing 2
ORAS	OTULIN-Related Autoinflammatory Syndrome
OTULIN	OTU Deubiquitinase with Linear Linkage Specificity
PACNS	Primary Angiitis of the Central Nervous System
PAPA	Pyogenic Arthritis, Pyoderma gangrenosum and Acne syndrome
PCR	Polymerase chain reaction
PEPD	Peptidase D
PID	Primary Immunodeficiency
PLAID	PLCG2-associated Antibody Deficiency and Immune Dysregulation
PLCG2	Phospholipase C Gamma 2
POLA1	DNA Polymerase Alpha 1, catalytic subunit
PRR	Pattern Recognition Receptors
PSTPIP1	Proline-Serine-Threonine Phosphatase Interacting Protein 1
RAB27A	RAS-Associated Binding protein 27A
RAG1/2	Recombination Activating Gene 1 and 2
SCC	Squamous cell carcinoma
SCID	Severe Combined Immunodeficiency
SCIG	Subcutaneous Immunoglobulin
SPINK5	Serine Peptidase Inhibitor Kazal Type 5
STAT1	Signal Transducer and Activator of Transcription 1
STAT3	Signal Transducer and Activator of Transcription 3
T cells	T lymphocytes
TACI	Transmembrane Activator and CAML Interactor
Th17	T helper 17 cells
TLR7	Toll-Like Receptor 7
UNC93B1	UNC93 homolog B1
USB1	U6 SnRNA Biogenesis 1
UV	Ultraviolet
VZV	Varicella zoster virus
WAS	Wiskott–Aldrich Syndrome protein
WHIM	Warts, Hypogammaglobulinemia, Infections, Myelokathexis syndrome
XRPD	X-linked Reticulate Pigmentary Disorder
